# Do Ultraconservative Access Cavities Hinder Endodontic Reintervention in Mandibular Incisors? A Laboratory Investigation

**DOI:** 10.1155/2024/5516067

**Published:** 2024-02-03

**Authors:** Caroline Meurer Luiz, Taynara Santos Goulart, Ketillyn da Silva Magalhães, Gilmar da Rosa Souza Junior, Lucas da Fonseca Roberti Garcia, Josiane de Almeida

**Affiliations:** ^1^Department of Endodontics, University of Southern Santa Catarina (UNISUL), Palhoça, Santa Catarina, Brazil; ^2^Department of Dentistry, Endodontics Division, Federal University of Santa Catarina (UFSC), Florianópolis, Santa Catarina, Brazil

## Abstract

This study assessed the influence of the type of endodontic access cavity on endodontic reintervention. Twenty mandibular central incisors were distributed into two groups (*n* = 10): TradAC group—traditional access cavities and UltraAC.Inc group—ultraconservative access cavities. After endodontic access, the root canals were prepared and obturated by the single cone technique. The filling material was removed with the Reciproc R25 instrument, followed by reinstrumentation with the R40 instrument. Images acquisition of each root canal hemisection was performed in a stereomicroscope to quantify the amount of remaining filling material. The amount of remaining filling material attached to the root canal walls was expressed in square millimeter (mm^2^). Data were statistically analyzed (one-way ANOVA and post hoc Student's *t*-tests). There was no statistically significant difference between TradAC and UltraAC.Inc groups (*p* > 0.05). None of the tested endodontics' access showed root canal walls completely free of filling material. Ultraconservative access cavities in mandibular incisors had no negative impact on the filling material removal.

## 1. Introduction

Adequate endodontic access should allow for the location, chemical–mechanical preparation, and obturation of the root canal [1]. Ultraconservative access cavities (UltraAC) aim to preserve part of the pulp chamber roof and pericervical dentin to increase the mechanical resistance of the endodontically treated tooth [2]. However, UltraAC may frequently hamper the chemical–mechanical preparation, resulting in the maintenance of contaminated areas, and leading to failure of the primary endodontic treatment [3–5].

The first treatment option for persistent infection in the root canal system is nonsurgical endodontic reintervention, which consists of removing the filling material, followed by new chemical–mechanical preparation and obturation [6, 7]. Nevertheless, one of the biggest challenges of endodontic reintervention is the complete removal of the filling material of the primary endodontic treatment [8]. The remaining filling material attached to the root canal walls may harbor microorganisms and necrotic remnants, in addition to interfering with the adhesion of the new filling material [9].

Compared to traditional access cavities (TradAC), minimally invasive access cavities led to a higher percentage of root canal walls not touched by endodontic instruments [10]. Therefore, it may be hypothesized that in teeth with UltraAC, the removal of the filling material is limited due to the restricted action of the instruments in the root canal [10, 11]. The UltraAC, popularly known as the “ninja” access, in anterior teeth (UltraAC.Inc) is performed in the center of the incisal edge, parallel to the long axis of the tooth, conserving the pulp horns as much pericervical dentin and pulp chamber roof as possible [12].

Considering the divergent literature on the advantages and disadvantages of the different types of endodontic access cavities [3, 12–16] and the lack of studies assessing the influence of minimally invasive access cavities on the nonsurgical endodontic reintervention, this in vitro study sought to answer the following question: Do ultraconservative access cavities hinder the endodontic reintervention in mandibular incisors?

## 2. Materials and Methods

### 2.1. Sample Size Calculation

The sample size was estimated based on a previous study that compared traditional and minimally invasive access cavities [17]. The ANOVA (fixed effects, special, main effects, and interactions) statistical test was performed. The type of power analysis was set a priori (compute required sample size—*α*, effect size, and power). According to the parameters *α* = 0.05, effect size = 0.80, and 95% testing power, a sample size of 20 teeth, distributed between two experimental groups (*n* = 10), was established to allow a reasonable error distribution for statistical analyses. The G ^*∗*^Power software (version 3.1.9.6) (Heinrich-Heine-University Düsseldorf, Düsseldorf, Germany) was used for sample size calculation.

### 2.2. Specimens' Selection and Endodontic Access Cavities Preparation

Twenty freshly extracted sound mandibular incisors with comparable dimensions were used in this study. Digital radiographic images in both buccolingual and mesiodistal directions were obtained to properly establish the pulp chamber dimensions and the root canal length. Only teeth with pulp chamber height < 2 mm [18], complete rhizogenesis, a single and straight root canal with a total length of 20 mm, and no signs of internal resorptions and/or calcifications were selected. Next, the teeth were carefully inspected under magnifying lens (×4) (SteREO 31 Discovery, v12, Carl Zeiss, Jena, Germany). The diameters of the apical portion of the root canals were confirmed with a size 10 K-file. The diameter of the cervical portion of the root canals was also confirmed with the aid of a digital calliper (Starret 727, Starret, Itu, SP, Brazil). Root canals with a diameter < 1.5 mm were discarded. Teeth that did not meet the inclusion criteria were discarded from the final specimen pool. The final sample was selected from 50 mandibular incisors.

After disinfection by immersion in a 0.5% chloramine T solution for 48 hr and washing with running water for 24 hr, the selected teeth were stored individually in plastic containers with 10% formalin solution at 37°C until the experiments were performed.

The teeth were randomly distributed (https://www.random.org) into two experimental groups (*n* = 10), according to the type of endodontic access, as follows: TradAC group—traditional access cavities and UltraAC.Inc group—ultraconservative access cavities.

**Figure 1:**
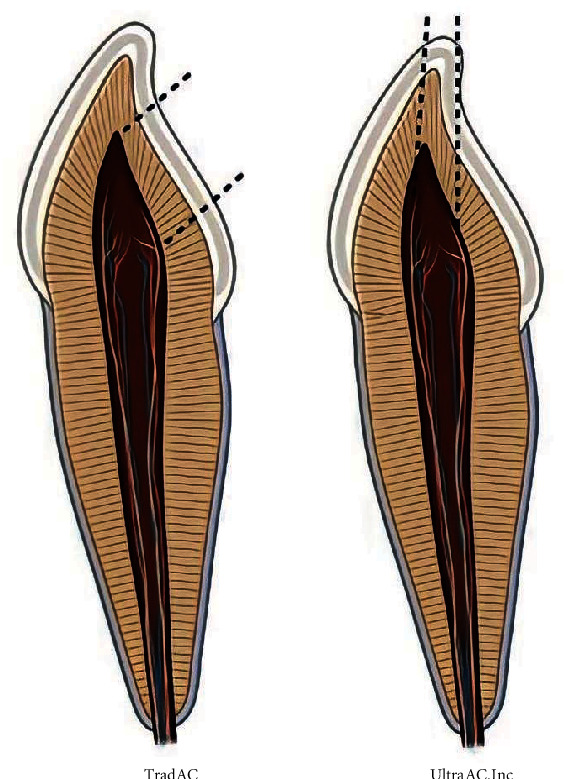
Endodontic access cavities: (a) traditional access cavity (TradAC) and (b) ultraconservative access cavity performed on the incisal edge (UltraAC.Inc). (Source: https://www.biorender.com/icon/primary-incisor-tooth-cross-section—modified by the authors).

All endodontic procedures, i.e., endodontic access cavities, root canal preparation, obturation, filling material removal, and reinstrumentation, were conducted under magnification with operative microscopy (DF Vasconcellos; Valença, RJ, Brazil) by a single operator, specialist in endodontics. TradAC was performed with high-speed diamond burs No. 1011 (spherical diamond bur—0.9 mm in diameter) and No. 3080 (conical diamond bur with inactive tip—1.2 mm in diameter at the most apical active portion) (American Burrs, Palhoça, SC, Brazil), according to the methodology described by Özkurt-Kayahan et al. [15]. The initial access point was performed on the lingual surface of the dental crown, 1 mm above the cingulum. The diamond bur No. 1011 was positioned perpendicular to the long axis of the tooth, with an inclination of 45°, until the pulp chamber was reached. The cavity was extended in the cervical–incisal and mesiodistal directions until the pulp chamber roof was completely removed. Afterward, the pericervical dentin was partially removed in the lingual region to establish direct access to the root canal (Figure 1(a)). Following the methodology described by Vieira et al. [19], UltraAC.Inc was performed with diamond bur No. 1011 (American Burrs), which was positioned just below the center of the incisal edge on the lingual surface of the crown, parallel to the long axis of the tooth until the pulp chamber was reached. The cavity was not extended, thus preserving the pericervical dentin and part of the pulp chamber roof (Figure 1(b)). For both groups, after access was completed, the initial exploration of the root canals was performed with a size 10 K-file (Dentsply-Maillefer, Ballaigues, Switzerland). The working length (WL) was set 1 mm below the total length of the tooth (19 mm).

### 2.3. Root Canal Preparation and Obturation

The root canals were mechanically prepared with a single-file reciprocating system (R25—25/.08) (Reciproc; VDW GmbH, Munich, Germany), coupled to a 6 : 1 reducing contra-angle (VDW Silver Reciproc, Sirona Dental Systems GmbH, Bensheim, Germany). The electric motor (VDW Silver Reciproc, Sirona Dental Systems) was programed in the “Reciproc All” function, and the root canal was prepared by thirds, with pecking motion to the apical direction, with a maximum amplitude of 3 mm, until reaching the WL. The patency of the apical foramen was passively maintained with a 10 K-file (Dentsply-Maillefer). Each time, the instrument was removed from the root canals, its active part was cleaned with a gauze soaked in 70% ethanol, and the root canals were irrigated with 5 mL of 2.5% NaOCl solution (Asfer Indústria Química, São Caetano do Sul, SP, Brazil) using NaviTip 30 ga needle (Ultradent) positioned 2 mm below the WL. The final irrigation was performed with 3 mL of 17% EDTA (Biodinâmica, Ibiporã, PR, Brazil), followed by 3 mL of 2.5% NaOCl solution (Asfer Indústria Química), 3 min for each solution, and finally neutralized with 5 mL of saline solution.

The root canals were then dried with absorbent paper cones (Dentsply-Maillefer) and filled by the single-cone technique. Initially, an epoxy resin-based root canal sealer (AH Plus; Dentsply Tulsa Dental Specialties, Tulsa, OK, USA) was mixed according to the manufacturer's recommendation and applied to the root canal walls using a gutta-percha cone. Then, a 25/.08 master gutta-percha cone (Reciproc; VDW GmbH) was coated with the sealer and inserted into the root canal. The excess of gutta-percha was removed up to 1 mm below the cementoenamel junction using a preheated plugger (Buchanan Plugger; SybronEndo Corporation, Orange, CA, USA). Then, the pulp chamber was cleaned with sponges soaked with 70% ethanol. Periapical radiographs of both buccolingual and mesiodistal directions were taken to certify the adequate filling of the root canals. Specimens with voids or gaps between the sealer and the gutta-percha cone and/or the filling material and root canal walls were discarded and replaced. The pulp chamber was sealed with temporary restorative material (Citodur; DoriDent, Wien, Austria). Next, the specimens were individually stored in closed plastic flasks containing distilled water to avoid dehydration, at 37°C, for 14 days to allow complete sealer's setting.

### 2.4. Endodontic Reintervention

Initially, the temporary restorative material was carefully removed so that the shape of the access cavities was not altered. Then, the filling material was removed with an R25 (25/.08) instrument from the Reciproc system (VDW GmbH). Removal was performed by thirds, with back-and-forth movements and minor pressure to the apical direction, with a maximum amplitude of 3 mm. The procedure was performed until reaching the WL and/or until there was no more evidence of filling material in the reflux of the irrigating solution or the active part of the instrument. Reinstrumentation of the root canal was performed with the R40 (40/.06) instrument (Reciproc; VDW), also by thirds, with back-and-forth movements and minor pressure to the apical direction, with a maximum amplitude of 3 mm, following the manufacturer's recommendations. At each instrument removal from the root canal for cleaning with a gauze soaked in 70% alcohol, the root canals were irrigated with 2 mL of 2.5% NaOCl solution (Asfer Indústria Química) using a NaviTip 30 ga needle (Ultradent). Each R25 and R40 instrument was used for filling material removal and reinstrumentation, respectively, of only one root canal.

### 2.5. Root Canal Filling Removal Analysis

**Figure 2:**
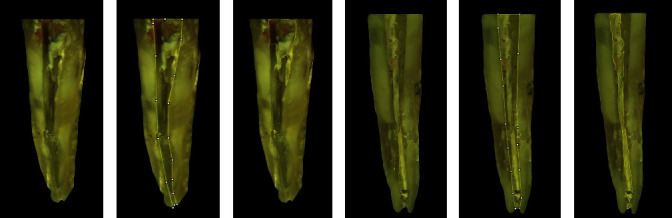
Representative images of the specimen from the TradAC group: (a, d) root hemisection; (b, e) external contour of the root canal space; and (c, f) delimitation of areas of the root canal containing remnants of filling material attached to the dentinal walls.

**Figure 3:**
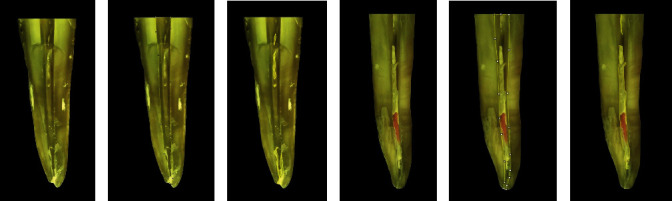
Representative images of the specimen from UltraAC.Inc group: (a, d) root hemisection; (b, e) external contour of the root canal space; and (c, f) delimitation of areas of the root canal containing remnants of filling material attached to the dentinal walls.

To evaluate the remaining filling material, lateral grooves were created with a spherical bur in the mesial and distal surfaces of the roots. Then, the teeth were carefully cleaved into two halves in the mesiodistal direction using a Lecron spatula (Quinelato, Rio Claro, SP, Brazil) to avoid the dislodgement of the remaining filling material from the root canal walls [20]. Images acquisition was performed in stereomicroscope (SteREO 31 Discovery, v12, Carl Zeiss, Jena, Germany), equipped with a digital camera (Sony Cyber-shot DSC-W530, Sony Brazil, São Paulo, SP, Brazil), and specific software (ZEISS Axio Vision, A1 and ZEM core v2.0.66.1000), under ×8 magnification, which provided a full view of each half of the teeth (Figures 2 and 3). The outer contour of each root canal hemisection (Figures 2(b), 2(e), 3(b), and 3(e)) and the areas corresponding to the remaining filling material (Figures 2(c), 2(f), 3(c), and 3(f)) were delineated with the ImageJ software (https://imagej.nih.gov/ij/index.html) using the following commands and plugin, respectively, “Analyze > Tools > Grid” and “Draw line or point grids.” At each specimen analysis, the software was calibrated with the aid of a ruler to allow a standard and reliable measurement. The analysis was carried out by an experienced endodontist, previously and properly trained regarding the software features, and calibrated to differentiate the remaining filling material from the root canal walls.

Before performing the measurements, the examiner underwent training on the software functionalities. Prior to the specimens assessment, the examiner also underwent calibration, involving the presentation of images depicting teeth with remaining filling material attached to the root canal walls. It is important to note that the images used for training and calibration were not used in the subsequent assessment. A maximum of 10 images were assessed daily, with an interval of 24 hr between each session of analysis. In order to minimize the risk of bias, 1 month after performing the measurements, the images were reassessed. No significant discrepancies were observed between the two periods of analysis. The intraexaminer agreement was considered excellent. Furthermore, the whole analysis was performed blindly. The total area of the root canal and the amount of remaining filling material attached to the root canal walls were expressed in square millimeter (mm^2^). The data obtained in square millimeter (mm^2^) were transformed into a percentage [20].

### 2.6. Statistical Analysis

The dataset had a normal distribution (Shapiro–Wilk test, *p*  > 0.05). The one-way analysis of variance was initially applied to the data (amount of remaining filling material) for comparison between experimental groups and complemented by the post hoc Student's *t*-test (*p*  < 0.05). All statistical tests were performed with a cut-off for significance at 5%. The SPSS software version 21.0 (IBM, Armonk, NY, USA) was used to perform the statistical analyses.

## 3. Results

No specimens were lost during the experiment. The mean values of root canal walls free of remaining filling material and their statistical comparison can be seen in Tables 1 and 2, respectively. None of the experimental groups had complete filling material removal. There was no statistically significant difference between TradAC and UltraAC.Inc groups (*p*  > 0.05). However, 30% of the filling material remained attached to the root canal walls.

## 4. Discussion

Minimally invasive endodontic access cavities aim to increase the fracture resistance of endodontically treated teeth [12, 13]. However, most of the laboratory studies conducted so far did not demonstrate favorable findings for these types of accesses [4, 15, 18]. In addition, in cases where nonsurgical endodontic reintervention is required, minimally invasive accesses may hinder the removal of filling material and reinstrumentation of the root canal [18, 19].

Bearing this gap in the literature in mind, the purpose of this in vitro study was to evaluate if ultraconservative access cavities indeed hinder endodontic reintervention in mandibular incisors. According to the results obtained herein, the formulated hypothesis was rejected since the different types of endodontic access tested allowed similar filling material removal during endodontic reintervention.

Among the methods used to quantify the remaining filling material after endodontic reintervention, the analysis of longitudinally cleaved teeth under a stereomicroscope is considered an eligible method, with accurate and reliable parameters [20, 21]. However, the use of this technique has limitations when compared to microcomputed tomography (micro-CT) which provides a 3D-analysis of the root canal [22]. Although studies using stereomicroscopes had a smaller impact, the utilization of micro-CT is limited due to its expensive nature, which hinders accessibility [21, 22]. Additionally, the scanning process of specimens is time consuming, and there is a possibility of generating artifacts [21, 22]. Despite the constraints associated with a 2D method, the stereomicroscope continues to be widely utilized due to its unique advantages [22]. It enables direct observation of specimens, providing an unadulterated view without the distortions and artifacts often encountered in radiographic and tomographic methods [20, 21]. For this reason, this method of analysis was used in the present study.

Divergences in the literature are widely found regarding the advantages and disadvantages of the different forms of endodontic access cavities [3, 4, 12, 13, 15, 16, 23]. Discrepancies in the methods presented by in vitro studies, such as the teeth selected and their anatomical characteristics [24], as well as the instrumentation systems used for the mechanical preparation [24], must be considered.

Teeth with comparable dimensions as possible (pulp chamber and root canals) were selected for the present study. Mandibular incisors have oval-shaped canals, which is a challenge for dental professionals [17]. Nevertheless, mandibular incisors usually have reduced dimensions and rarely present sharp curvatures [17], which may have contributed to the similar performance of TradAC and UltraAC.Inc experimental groups. Previous studies that also used mandibular incisors demonstrated that the different endodontic access cavities did not influence the percentage of root canal walls not touched by the instruments [17, 19]. The larger diameter instrument (R40) used in this study for reinstrumentation of the root canals may have contributed to the more effective removal of the remaining filling material, even in the UltraAC.Inc experimental group [16].

Conversely, other studies have shown that in molar teeth, minimally invasive access cavities led to higher percentages of root canal walls not touched by instruments than TradAC [2, 25]. Furthermore, in premolars, minimally invasive access cavities resulted in a greater amount of remaining filling material attached to the root canal walls after endodontic reintervention [8].

In this study, all specimens had remaining filling material attached to the root canal walls after reinstrumentation, regardless of the type of endodontic access cavity. These results corroborate studies that have demonstrated that no endodontic reintervention protocol may completely remove the filling material from the root canal walls [26]. Preceding studies have also demonstrated the detrimental impact of using solvent solutions, such as chloroform, for filling material removal [27]. In addition to the high toxicity levels of solvent solutions, the softened gutta-percha strongly adheres to the root canal walls, making its removal even more difficult [27]. For this reason, no solvents were used herein.

The major operative difficulties of minimally endodontic access cavities are the initial location of the root canals and the cleaning of the pulp chamber postobturation [16, 18]. In the present study, all procedures were performed under magnification with operative microscopy, which significantly helped in locating the root canal entrance, both in primary endodontic treatment, as well during endodontic reintervention. Moreover, the cleaning of the postobturation pulp chamber also benefited from the use of the operating microscope.

Previous research has reported the possibility of performing successful root canal preparation in teeth with minimally invasive access cavities [8, 12], especially when using thermally treated NiTi instruments. Instruments of different diameters and tapers of the Reciproc system were used to remove the filling material and for reinstrumentation of the root canal, i.e., R25 and R40, respectively. Instruments from the Reciproc system have shown excellent results in cases of endodontic reintervention, which may explain the results found in the present research. Reciproc instruments are manufactured from M-Wire NiTi alloy, which exhibits greater flexibility and resistance to cyclic fatigue [7, 28]. Furthermore, these instruments can produce lesser apical transportation and preserve the WL in comparison with instruments manufactured from conventional NiTi alloys [7, 28]. M-Wire instruments, such as those from the Reciproc system, are the most adequate choice for root canal preparation of teeth with UltraAC, with a greater percentage of instrumented root canal walls and lower levels of apical transportation [7, 28].

Further research should be carried out using the same methodological design of the present study, however, involving different instrumentation systems and different groups of teeth to consolidate the advantages and limitations of minimally invasive access cavities in endodontic reintervention.

## 5. Conclusions

In conclusion, within the limitations of this study, ultraconservative access cavities in mandibular incisors had no negative impact on the removal of the filling material during endodontic reintervention. A high amount of filling material remained attached to the root canal walls after reintervention in both groups.

## Figures and Tables

**Table 1:** Mean values (%) and standard deviation (±) of root canal walls free of remaining filling material.

TradAC	UltraAC.Inc
69.10 ± 13.45^a^	72.10 ± 15.19^a^

Lowercase letter in line means significant statistical difference (*p* < 0.05).

**Table 2:** Student's *t*-test for the experimental groups.

Student's *t*-test									
	Levene test for equality of variances		*t*-test for equality of means						
	*F*	Sig.	*t*	df	Sig. (extremities)	Mean difference	Standard error of difference	95% confidence interval of difference	
								Inferior	Superior
Equal variances assumed	0.813	0.377	0.499	21	0.623	2.99633	6.00594	−9.49371	15.48637
Equal variances not assumed			0.502	20.981	0.621	2.99633	5.97293	−9.42575	15.41841

## Data Availability

The data that support the findings of this study are available from the corresponding author upon reasonable request.
